# Surgical outcomes of anterior lumbar interbody fusion in revision lumbar interbody fusion surgery

**DOI:** 10.1186/s13018-023-03972-6

**Published:** 2023-07-13

**Authors:** Cheng-Min Shih, Cheng-En Hsu, Kun-Hui Chen, Chien-Chou Pan, Cheng-Hung Lee

**Affiliations:** 1 Department of Orthopaedic Surgery, Taichung Veterans General Hospital, 1650 Taiwan Boulevard Sect. 4, Taichung, 40705 Taiwan; 2 College of Biological Science and Technology, National Yang Ming Chiao Tung University, Hsinchu, Taiwan; 3 Department of Physical Therapy, Hungkuang University, Taichung, Taiwan; 4 Sports Recreation and Health Management Continuing Studies-Bachelor’s Degree Completion Program, Tunghai University, Taichung, Taiwan; 5 Department of Post-Baccalaureate Medicine, College of Medicine, National Chung Hsing University, Taichung, Taiwan; 6 College of Computing and Informatics, Providence University, Taichung, Taiwan; 7 Department of Rehabilitation Science, Jenteh Junior College of Medicine, Nursing and Management, Miaoli, Taiwan; 8 Department of Food Science and Technology, Hungkuang University, Taichung, Taiwan

**Keywords:** Anterior lumbar interbody fusion, Revision lumbar interbody fusion, Nonunion, Lumbar fusion, Revision spine surgery

## Abstract

**Backgrounds:**

Anterior lumbar interbody fusion (ALIF) is an attractive option for revision lumbar interbody fusion as it provides wide access for implant removal and accommodation of large interbody grafts for fusion. However, revision lumbar interbody fusion surgery has not been found to result in significantly better functional outcomes compared with other approaches. To date, no prognostic factors of anterior lumbar interbody fusion in revision lumbar interbody fusion have been reported. In this study, we investigated the surgical results and possible prognostic factors of anterior lumbar interbody fusion in revision lumbar interbody fusion.

**Methods:**

Patients who received revision interbody fusion surgery between January 2010 and May 2018 in our hospital were reviewed. Clinical outcomes were determined according to whether the VAS score improvement in back pain and leg pain reached the minimum clinically important difference (MCID) and Macnab criteria. Radiographic outcomes were assessed with fusion rate, preoperative, and postoperative lumbar lordosis. Operative-relative factors that may affect clinical outcomes, such as BMI, existence of cage migration, cage subsidence, pseudarthrosis, previous procedure, and number of fusion segments, were collected and analyzed.

**Results:**

A total of 22 consecutive patients who received ALIF for revision interbody fusion surgery were included and analyzed. There were 9 men and 13 women with a mean age at operation of 56 years (26–78). The mean follow-up was 73 months (20–121). The minimal clinically important difference (MCID) was reached in 11 (50%) of the patients for back pain and 14 (64%) for leg pain. According to the modified Macnab criteria, 73% of the patients in this study had successful outcomes (excellent or good). The pain and lumbar lordosis had significant improvement (*P* < 0.05). Preoperative fusion segment ≥ 2 was shown to be a poor prognostic factor for back pain improvement reaching MCID (*P* = 0.043).

**Conclusions:**

ALIF has proven effective for revision lumbar fusion surgery, yielding positive clinical and radiographic results. However, having two or more preoperative fusion segments can negatively impact back pain improvement.

*Level of evidence*: IV.

## Backgrounds

Transforaminal lumbar interbody fusion (TLIF) and posterior lumbar interbody fusion (PLIF) are very common spinal fusion procedures. However, complications such as segmental pseudarthrosis or migration of the interbody cage could cause persistent pain or new onset neurologic symptoms [1, 2]. Retrieval of interbody cage is usually necessary in the revision surgery. However, the removal procedure from the posterior approach is challenging due to the high risk of neurologic injury, with both the superior and the inferior vertebral body end plates leading to subsequent graft subsidence [3–7]. Anterior lumbar interbody fusion (ALIF) is an attractive option for revision surgery of pseudarthrosis since it avoids working through scar, provides wide access for implant removal to the disk space, and accommodates large interbody grafts with a substantial surface area for fusion, which also restores the sagittal balance of the spine [5, 6, 8, 9].

Despite these advantages, ALIF has shown no significantly better functional outcomes compared to other approaches [10, 11]. Little attention has been paid to the prognostic factors that affect surgical outcomes. In this study, we investigated surgical outcomes and possible prognostic factors for anterior lumbar interbody fusion in revision lumbar interbody fusion.

## Methods

### Patient enrollment

Patients who received revision interbody fusion surgery between January 2010 and May 2018 in our hospital were identified and considered for enrollment.

The inclusion criteria were normal mental health status and a complete dataset comprising all functional status questionnaires and measurements performed in this study. The exclusion criteria were pathologic fractures, infection, or previous anterior approach spine surgery.

### Data collection

Patients’ demographics including age, gender, BMI, and symptom duration were collected. Surgical information including level of implanted cages, preoperative cage migration, cage subsidence, pseudarthrosis, previous procedure, fusion segments, lumbar lordosis, and follow-up were collected.

All patients had preoperative radiographs, computed tomography (CT) scans, or magnetic resonance imaging (MRI) of the lumbar spine. Dynamic lumbar spine radiography was done to confirm the diagnosis of cage migration, pseudarthrosis, subsidence, and fusion success. The determination of fusion success was independently assessed by a blinded radiologist according to the following criteria: the absence of motion between the fusion segments on lateral flexion–extension views, no radiolucency in the disk space, and formation of a bone bridge connecting the vertebral bodies above and below.

Clinical outcomes were graded using the visual analog scale (VAS; score range 0–10, with 0 reflecting no pain) and Macnab criteria. The percentage of patients reaching the minimum clinically important difference (MCID) was examined for each group. This measure represents the minimum improvement in an outcome measure in which the patient perceives a worthwhile benefit [12–14]. The MCID was defined as 1.2-point decrease in back pain, and a 1.6-point decrease in leg pain based on previously published thresholds [15].

All radiographic data were collected by a blinded observer who had 7 years’ experience performing spinal surgery. Complications were identified through medical records and classified as intraoperative or postoperative (≤30 day of surgery). Intraoperative complications included vascular injury (arterial or venous), ureter, dural, or bowel injury. Postoperative complications included wound complications (dehiscence, hernia, or infection), hematoma (retroperitoneal or rectus sheath), screw loosening, cage migration, neurologic deficit, or medical—classified as pulmonary (pneumonia, pleural effusion, pulmonary edema, or respiratory failure), and cardiac (arrhythmia, congestive heart failure, or myocardial infarction).

## Surgical technique

In this study, all of the cages were removed using the anterior retroperitoneal approach with anterior cantilever technique [16]. All patients received pedicle screw-based posterior instrumentation during the previous operation, except for 4 patients who underwent cage-stand-alone surgery in the previous operation. For the 18 patients with posterior instrumentation, all of the existing pedicle screws and rods were removed first, and the wound was closed temporarily. After removal of previous posterior instrumentation, the patients were repositioned in the supine position with oxygen saturation probe on the ipsilateral great toe to monitor perfusion. A lateral radiograph by C-arm was obtained for marking the target level; the abdomen was then prepared and draped following the standard sterile procedure. An 8 cm longitudinal median incision was made beneath the umbilicus. The rectus abdominis muscle was divided medially and retracted laterally to avoid pseudohernia from denervation. The psoas muscle and great vessels were then visualized and carefully retracted laterally. Blunt dissection was used to separate the peritoneum from the posterior rectus sheath. After the target level was identified, specific retractors for the anterior approach (SynFrame retractor system, Synthes GmbH, Switzerland) were used to maintain the exposure. The median sacral vessels were coagulated if necessary. The anterior longitudinal ligament and thickened fibrotic anterior annulus at the target level were incised. The residual disk was removed to expose the previously inserted cage. After removing the surrounding soft tissue and the remaining disc, a Cobb Elevator was used to distract the upper and lower endplates as a cantilever to increase the working space. In most cases of pseudarthrosis after TLIF or PLIF, the cage is loose and movable. Thus, these cages can be easily removed after removing surrounding soft tissue. If a cerebrospinal fluid (CSF) leak is identified, fibrin sealant (TISSEEL, Baxter International Inc, Deerfield, Illinois) can be injected prior to placement of the interbody implant.

After removal of the previous cage and completing meticulous endplate preparation, a trial cage was inserted temporarily to dilate the disk space. Then, a large, wedge-shaped lordotic design cage, could be symmetrically placed at the desired level. Finally, the position of the interbody cage and reduction was confirmed via C-arm fluoroscopy. After checking for bleeders and removal of retractors, the wound was closed layer by layer, and the patient was repositioned to the prone position for the final stage. The temporarily closed wound over the back was opened. The pedicle screws were inserted into the desired level, and two lordotic rods were placed in a suitable position. The pedicle screws and rods were carefully compressed to create lumbar lordosis. The screw position and lumbar spine alignment were confirmed via C-arm fluoroscopy; the posterior approach was then closed. Postoperative plain radiographs were obtained at regular intervals to assess interbody fusion and slip correction.

### Postoperative evaluation

Patients were followed up at two weeks, one month, three months, six months, one year, and two years after operation. Spinal lumbosacral orthosis application was used for postoperative 8 weeks.

### Statistical analysis

Data analysis was performed using SPSS software (Version 20.0; Chicago, Illinois). Univariate analysis was performed using frequencies for descriptive statistics. Normality of data distribution was assessed for continuous variables using Q-Q plots and the Shapiro–Wilk test. One way ANOVA was used in the analysis of continuous variables. Chi-square and Fisher’s exact test were used in the analysis of categorical variables. Wilcoxon signed rank test was used to compare for related data. Logistic regression was also performed to control for confounders and selection bias. Factors included age, gender, BMI, the number of fusion segments, previous procedures, symptom duration, cage migration, subsidence, and pseudarthrosis as these factors have been previously shown to influence outcomes after lumbar fusion surgery. Correlations were considered significant if p values were less than 0.05 (two-sided).

## Results

**Fig. 1:**
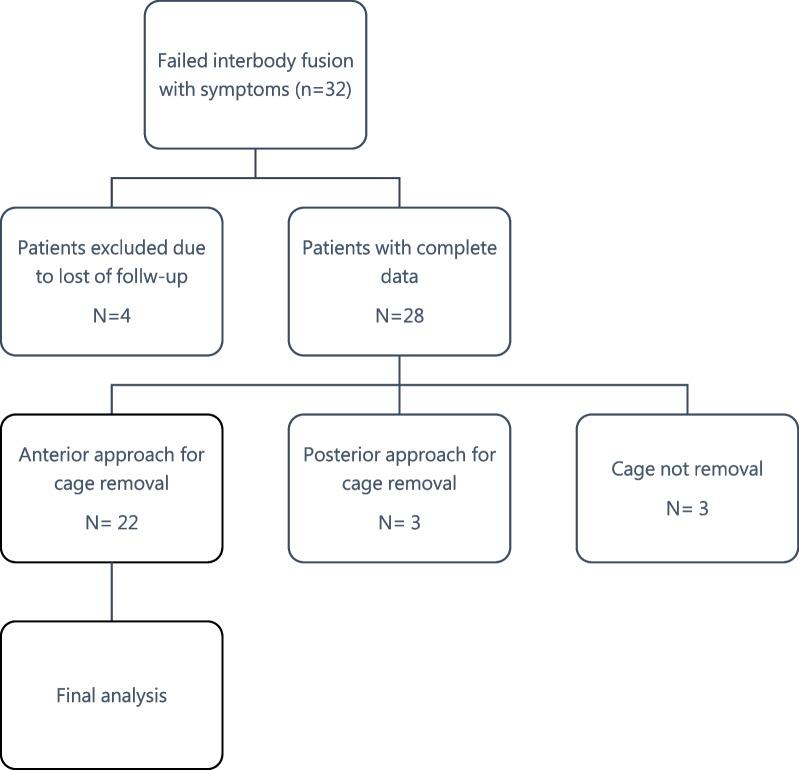
The flowchart of patient enrollment showing 22 patients who underwent anterior approach for cage removal was included in the analysis of the prognostic factors that affected outcomes (bold frame)

A total of 22 patients who received ALIF and posterior instrumentation for revision interbody fusion surgery were included and analyzed. The flowchart of patient enrollment is displayed in Fig. 1. There were 9 men and 13 women with a mean age at operation of 56 years (26–78). The mean follow-up was 73 months (range, 20–121 months). Minimal clinically important difference (MCID) was reached in 11 (50%) of patients for back pain and 14 (64%) for leg pain. The demographic data and intraoperative details are shown in detail in Table 1.

**Table 1:** Characteristics and intraoperative details of 22 patients undergoing ALIF for revision interbody fusion surgery

Characteristics	(*n* = 22)
Age, years, Mean ± SD (Range)	56 ± 13.7 (26–78)
Female Gender, *n* (%)	13 (59)
BMI ≥ 30, *n* (%)	9 (41)
Follow-up time, months, Mean ± SD, (Range)	73 ± 33.9 (20–121)
Complication, *n* (%)	1 (9)
Operation time, min, Mean ± SD (Range)	393 ± 93.0 (141–530)
Blood loss	
Anterior approach, ml, Mean ± SD, (Range)	66.4 ± 71.9 (10–300)
Posterior approach, ml, Mean ± SD, (Range)	687.7 ± 584.1 (30–2500)
Blood transfusion, *n* (%)	8 (36)
Fusion segments ≥ 2, *n* (%)	12 (55)
Previous procedure	
TLIF *n* (%)	15 (68)
PLIF *n* (%)	7 (32)
Symptom duration, months, Mean ± SD (Range)	25 ± 26.1 (2–96)
Cage migration, *n* (%)	11 (50)
Cage subsidence, *n* (%)	11 (50)
Pseudarthrosis	17 (77)
Interbody fusion device	
SynCage	18 (82)
TM cage	2 (9)
Bone graft	2 (9)
Back pain reached MCID	11 (50)
Leg pain reached MCID	14 (64)
Cage level	
L5-S1, *n* (%)	7 (32)
L4-L5, *n* (%)	10 (46)
L3-L4, *n* (%)	0 (0)
L2-L3, *n* (%)	2 (2)
≥ 2 cage, *n* (%)	3 (14)

*MCID* minimal clinically important difference

**Fig. 2:**
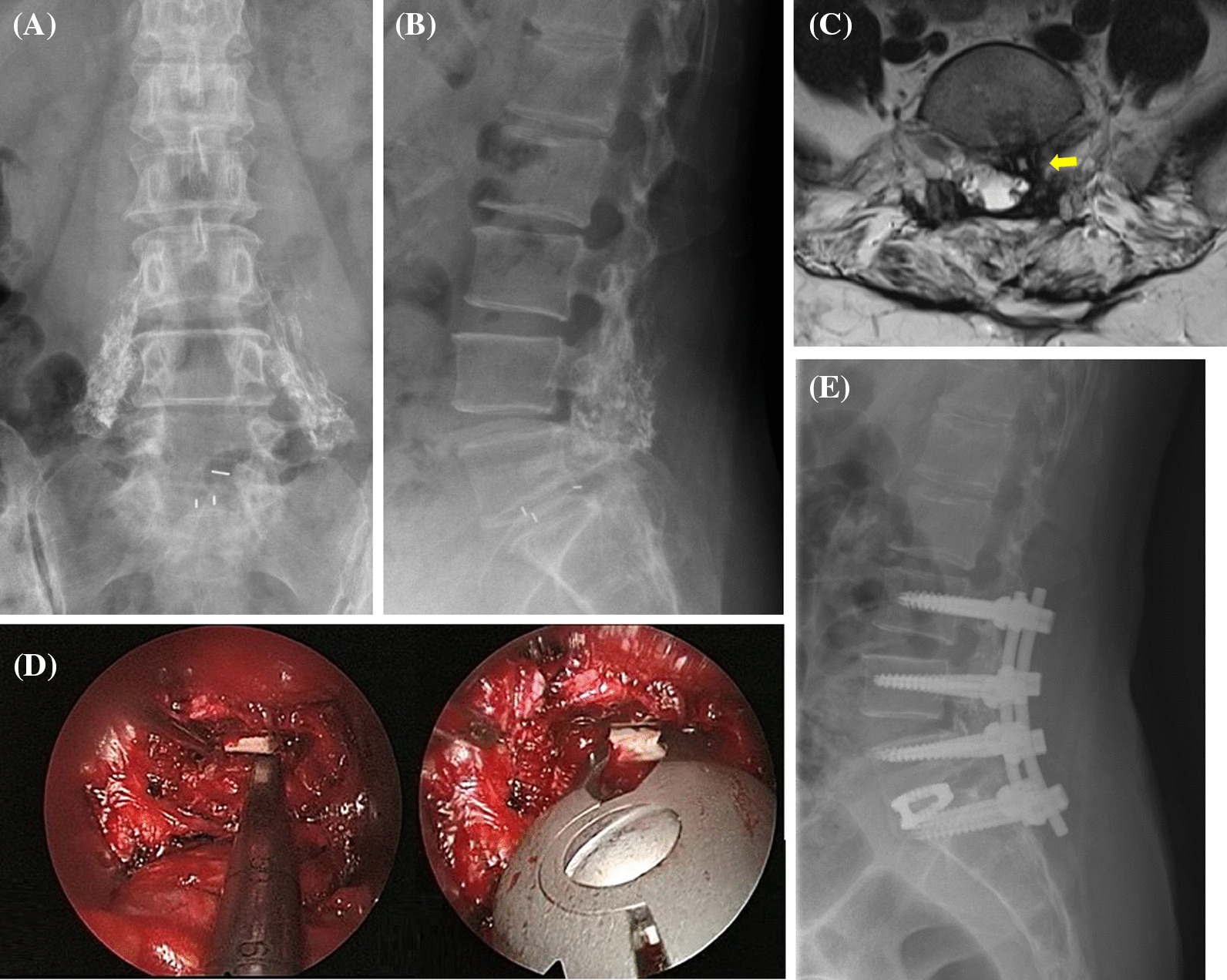
Anteroposterior (**A**) and lateral (**B**) lumbar spine radiography demonstrates a failed posterolateral fusion and pseudarthrosis of L5-S1 level. MRI (**C**) shows posterior migration of cage with root compression (yellow arrow). Intraoperative endoscopy picture (**D**) shows that the cage can be loosened and retrieved with forceps. Postoperative lateral lumbar spine radiography (**E**) shows good cage position and lumbar spine alignment after ALIF with posterior instrumentation

The results of the analysis of prognostic factors affecting whether patients’ back pain improvement reached MCID or not are shown in Table 2. Among the 12 patients who had preoperative fusion segments ≥ 2, nine (82%) patients did not reach MCID; however, in 10 patients who had preoperative fusion segments < 2, only two (18%) patients did not reach MCID. The rate of treatment failure for patients with preoperative fusion segment ≥ 2 was significantly higher than in those with preoperative fusion segment < 2 (*p* = 0.03, Fisher’s exact test). Other variables (age, gender, BMI, previous procedure, symptom duration, cage migration, cage subsidence, and pseudarthrosis) did not have a significant association with the clinical outcome of back pain improvement (Fig. 2).

**Table 2:** Analysis of prognostic factors affect patients’ clinical outcomes in back pain

	All (*n* = 22)	Improvement 2 (*n* = 11)	No improvement (*n* = 11)	P value
Age, years, Mean ± SD	56 ± 13.7	58 ± 13.3	54 ± 14.2	0.434
Female gender, *n* (%)	13 (59)	6 (60)	7 (59)	1.000
BMI ≥ 30, *n* (%)	7 (41)	2 (18)	5 (46)	0.361
Preoperative fusion segments				
< 2, *n* (%)	10 (46)	8 (73)	2 (18)	0.030
≥ 2, *n* (%)	12 (45)	3 (27)	9 (82)	
Previous procedure				
TLIF *n* (%)	15 (68)	7 (64)	8 (73)	1.000
PLIF *n* (%)	7 (32)	4 (36)	3 (27)	
Symptom duration, months, Mean ± SD	25 ± 26.1	26 ± 27.2	24 ± 2.6.4	0.878
Cage migration, *n* (%)	11 (50)	5 (46)	6 (55)	1.000
Cage subsidence, *n* (%)	11 (50)	5 (46)	6 (55)	1.000
Pseudarthrosis	17 (77)	9 (82)	8 (73)	1.000

The results of the analysis of prognostic factors affecting whether patients’ leg pain reached MCID or not are shown in Table 3. No variables (age, gender, BMI, preoperative fusion segments, previous procedure, symptom duration, cage migration, cage subsidence, and pseudarthrosis) were significantly associated with the clinical outcome of leg pain improvement.

**Table 3:** Comparison of patients’ outcomes according to whether leg VAS reached MCID

	All (*n* = 22)	Improvement (*n* = 14)	No improvement (*n* = 8)	P value
Age, years, Mean ± SD	56 ± 13.7	58 ± 12.6	51 ± 14.9	0.204
Female gender, *n* (%)	13 (59)	9 (64)	4 (50)	0.662
BMI ≥ 30, *n* (%)	7 (32)	3 (38)	4 (29)	0.510
Fusion segments ≥ 2, *n* (%)				
≥ 2, *n* (%)	12 (55)	9(64)	3 (38)	0.378
< 2, *n* (%)	10 (45)	5 (36)	5 (62)	
Previous procedure				
TLIF *n* (%)	15 (68)	9 (64)	6 (75)	1.000
PLIF *n* (%)	7 (32)	5 (36)	2 (25)	
Symptom duration, months, Mean ± SD	25 ± 26.1	27 ± 26.9	23 ± 26.4	0.746
Cage migration, *n* (%)	11 (50)	8 (57)	3 (38)	0.659
Cage subsidence, *n* (%)	11 (50)	7 (50)	4 (50)	1.000
Pseudarthrosis	17 (77)	11 (79)	6 (75)	1.000

In the multivariate regression analysis of poor improvement in back pain after the ALIF revision interbody fusion surgery, preoperative fusion segment ≥ 2 was found to have a statistically significant effect on poor back pain improvement after multivariate adjustment (*P* = 0.0435) (Table 4).

**Table 4:** Multivariant regression of prognostic predictors for poor outcomes in back pain improvement after ALIF for revision interbody fusion

Risk factors	Adjusted OR (95% C.I.)	P value
Age, years	1.034 (0.939–1.137)	0.497
Gender		
Male	1.00 (Ref.)	
Female	0.575 (0.038–8.758)	0.690
BMI		
< 30	1.00 (Ref.)	
≥ 30	2.468 (0.159–38.223)	0.518
Fusion segments		
< 2	1.00 (Ref.)	
≥ 2	10.714 (1.073–106.948)	0.043
Previous procedure		
TLIF	1.00 (Ref.)	
PLIF	0.593 (0.041–8.628)	0.702
Symptom duration	2.457 (3.765–36.140)	0.561
Cage migration		
No	1.00 (Ref.)	
Yes	1.083 (0.092–12.746)	0.949
Pseudarthrosis		
No	1.00 (Ref.)	
Yes	0.377 (0.010–13.977)	0.597
Cage subsidence		
No	1.00 (Ref.)	
Yes	2.269 (0.130–32.938)	0.607

Table 5 demonstrates the changes in back and leg VAS pain scores, as well as lumbar lordosis after the revision surgery. Significant enhancements in back and leg VAS pain scores, L1-S1 lordosis, L4-S1 lordosis, and segmental lordosis were noted (*P* < 0.001, < 0.0010, 0.01, 0.002, and < 0.001, respectively).

**Table 5:** Alteration in VAS pain score and Lumbar Spine Lordosis in Revision Surgery via Anterior Lumbar Interbody Fusion (ALIF)

Lumbar lordosis, Mean ± SD	Preoperative	Postoperative	P value
VAS pain score			
For back, Mean ± SD (range)	7.4 ± 1.0 (5–9)	4.0 ± 1.2 (1–6)	< 0.001
For leg, Mean ± SD (range)	7.7 ± 0.8 (6–9)	2.0 ± 1.4 (0–6)	< 0.001
L1-S1 lordosis, Mean ± SD (range)	33 ± 13.3 (10–65)	38 ± 13.8 (7–66)	0.01
L4-S1 lordosis, Mean ± SD (range)	22 ± 9.0 (0.5–39)	26 ± 9.4 (4–42)	0.002
Segmental lordosis*, Mean ± SD (range)	14 ± 6.8 (4–39)	19.5 ± 7.2 (2–34)	< 0.001

^*^Segmental lordosis: the lordosis degree of operative segments

Table 6 presents the clinical results as per the modified Macnab criteria. A successful outcome (rated as excellent or good) was achieved by 73% of the patients in this study, with all patients demonstrating symptomatic improvement (ranging from excellent to fair). All of the 22 patients achieved interbody fusion in the reduced position within postoperative 9 months. No cases with subsidence required surgical revision. One patient complained of persistent back pain and S2 screw loosening was found at 2 months postoperatively. Two additional months of back brace wearing was suggested for this patient who achieved uneventful solid fusion 2 months later. No cage dislodgement or other type of implant failure occurred in our series.

**Table 6:** Clinical outcomes of 22 patients according to modified Macnab criteria

Result	Patients (*n*)	Rate (%)	Criteria
Excellent	4	18	No pain; no restriction of mobility; return to normal work and level of activity
Good	12	55	Occasional nonradicular pain; relief of presenting symptoms; return to modified work
Fair	6	27	Some improved functional capacity; still handicapped and unemployed
Poor	0	0	Continued objective symptoms of root involvement; additional operative intervention needed at the index level irrespective of length of postoperative follow-up

## Discussion

This is the first study to investigate surgical outcomes and prognostic factors for ALIF in revision interbody fusion surgery. The findings from our study indicate that ALIF is an efficient technique for lumbar fusion revision surgery, demonstrating favorable clinical and radiographic outcomes. Nonetheless, the presence of two or more fused segments prior to the surgery may adversely affect the improvement of back pain. This finding is important for operative planning and setting appropriate preoperative expectations in patients undergoing ALIF for revision interbody fusion surgery.

**Fig. 3:**
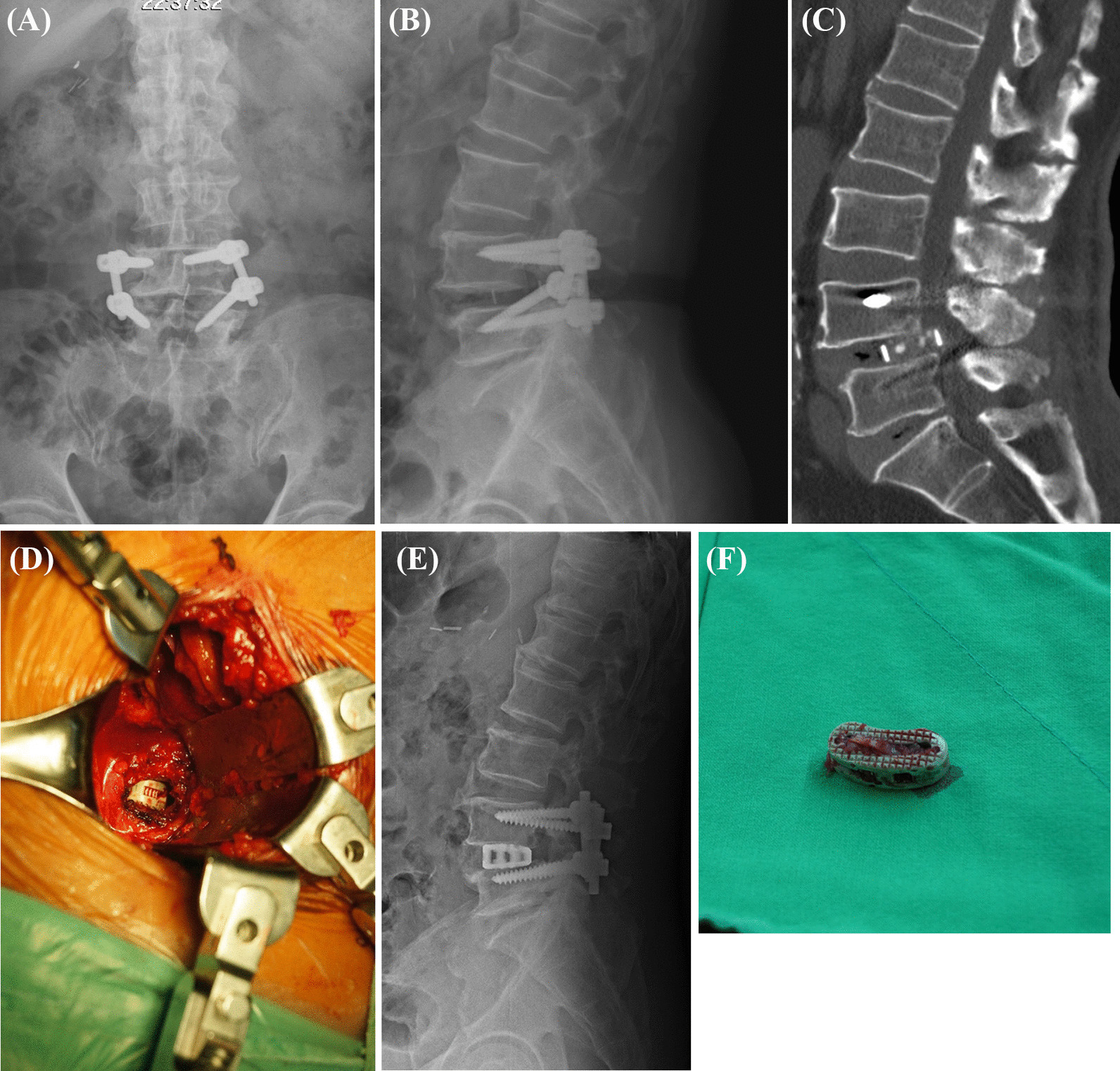
Anteroposterior (**A**) and lateral (**B**) lumbar spine radiography demonstrates cage posterior migration and screw malposition. Computed tomography (**C**) scan shows that the spinal cord was compressed by the posteriorly migrated cage. The position of the migrated cage can be easily reached via the anterior retroperitoneal approach (**D**). Postoperative lateral lumbar spine radiography (**E**) shows good cage position after ALIF with posterior instrumentation

In the ALIF surgical procedure, the anterior longitudinal ligament (ALL) is released to open the disk space, which makes it much easier to retrieve the cage compared with posterior approaches (Fig. 3). ALIF also has advantages from a biomechanical perspective to provide a larger region than PLIF and TLIF for fusion, which is especially important in revision surgery [9]. Despite its minimally invasive nature and very high successful fusion rate [5, 8, 17, 18], ALIF is still reported to result in poor to modest functional outcomes similar to those achieved with other posterior approaches in revision surgery [10, 11]. Owens et al. compared the functional outcomes of 128 patients who underwent posterolateral fusion (PSF), TLIF, ALIF alone, or combined anterior and posterior spinal fusion (AP) in revision spinal fusion surgery and found that type of surgical approach did not impact patient outcomes [10]. However, in their study, among the patients, the percentage of cases who had a failed interbody fusion requiring revision was not reported. Safaee et al. reviewed 84 cases undergoing ALIF with cage retrieval for pseudarthrosis after TLIF and reported a 97% fusion rate and a 22% complication rate. They found that older age and operation level above L3 were two risk factors for significantly higher complications in ALIF revision interbody fusion surgery [6]. There were only 2 patients who underwent ALIF revision interbody fusion surgery at operation above L3 in our study. No complication was found in these patients during our study period. In addition to complications as risk factors, we found that the existence of two or more fusion segments preoperatively was a poor prognostic factor for back pain improvement in the ALIF revision lumbar interbody fusion. Multilevel fusion is a common treatment for multilevel degenerative disease [19–21]. However, adverse outcomes associated with increasing fusion levels include higher complication rate, more frequent back pain, and worse work disability [22–24]. In the patient group with pseudarthrosis, those with multilevel fusion had a higher rate of concurrent sagittal imbalance, previous insufficient decompression, and adjacent disease [22, 24, 25]. In our protocol, all of the cages were retrieved via the anterior retroperitoneal approach. Posterior approach is usually for screw and rod revision, and posterior decompression is only performed in patients with preoperatively found stenosis. However, concurrent nerve compression and spinal stenosis are sometimes hard to detect in an imaging study due to artifacts from a previous implant. This may explain why patients with multilevel fusion levels had less back pain improvement after ALIF revision lumbar interbody fusion.

When cage malposition with nerve compression has occurred, fibrous tissue adherences around the cage increase the risk of dural tear during the cage extraction. Intraoperatively, the use of neuro-monitoring is important when cage retrieval is performed. In our study, no dural tear or nerve injury was noted during the operation. Revision of a mal-positioned cage may be very difficult in the posterior approach. New cage insertion from the posterior approach may subside into the previously fractured endplate. Anterior approach would be a reasonable alternative option to retrieve the cage. It provides a comfortable space to prepare the endplate. Placing a larger cage anteriorly avoids case subsidence and allows a larger fusion area as well as lumbar lordosis. An anterior approach should be considered first in the event of cage malposition with nerve compression if the access was not used before.

This study had several limitations and sources of bias that need to be addressed. First, due to the retrospective nature of this case series, we did not have a control group and some parameters that may have affected the clinical results, such as mental status and bone mineral density, were not included in our analysis. Second, the case number was small in our study, so some parameters did not reach statistical significance. Hence, future studies with larger case numbers are warranted to investigate the surgical effects of these parameters. Third, the neurophysiologic monitoring was not used in all cases, especially in the early period. Nonetheless, no patients developed neurological deficits in our study, and ALIF was reported to have a low possibility of neural injury during the operation [26]. However, neurophysiologic monitoring equipment is recommended during cage retrieval in revision interbody fusion surgery. Fourthly, CT myelography data were not included in this study. However, it is a highly useful tool for assessing concurrent nerve compression and spinal stenosis, particularly when artifacts from a previous implant might obstruct CT or MRI imaging. Future research on this topic should consider incorporating CT myelography in their methodologies.

## Conclusions

Our research demonstrates that ALIF is a successful approach for revision lumbar fusion surgery, producing encouraging clinical and radiographic outcomes. However, it is important to note that the presence of two or more preoperative fusion segments might hinder improvements in back pain.

## Data Availability

The data that support the findings of this study are available from the corresponding author upon reasonable request.

## Abbreviations

ALL: Anterior longitudinal ligament
ALIF: Anterior lumbar interbody fusion
PLIF: Posterior lumbar interbody fusion
TLIF: Transforaminal lumbar interbody fusion
MCID: Minimum clinically important difference
BMI: Body matrix index
LL: Lumbar lordosis
SVA: Sagittal vertical axis
